# High Birth Weight Increases the Risk for Bone Tumor: A Systematic Review and Meta-Analysis

**DOI:** 10.3390/ijerph120911178

**Published:** 2015-09-09

**Authors:** Songfeng Chen, Lin Yang, Feifei Pu, Hui Lin, Baichuan Wang, Jianxiang Liu, Zengwu Shao

**Affiliations:** 1 Department of Orthopedics, Union Hospital, Tongji Medical College, Huazhong University of Science and Technology, Wuhan 430022, China; E-Mails: chensongfeng123@126.com (S.C.); pufeifeiemail@163.com (F.P.); linhuimedicine@163.com (H.L.); wangbaichuan-112@163.com (B.W.); liujianxiangljx@163.com (J.L.); 2 Department of Pediatrics, Wuhan Medical Care Center for Women and Children, Wuhan 430016, China; E-Mail: yanglin2439@163.com

**Keywords:** birth weight, bone tumor, osteosarcoma, Ewing sarcoma of bone, chondrosarcoma, meta-analysis

## Abstract

There have been several epidemiologic studies on the relationship between high birth weight and the risk for bone tumor in the past decades. However, due to the rarity of bone tumors, the sample size of individual studies was generally too small for reliable conclusions. Therefore, we have performed a meta-analysis to pool all published data on electronic databases with the purpose to clarify the potential relationship. According to the inclusion and exclusion criteria, 18 independent studies with more than 2796 cases were included. As a result, high birth weight was found to increase the risk for bone tumor with an Odds Ratio (*OR*) of 1.13, with the 95% confidence interval (95% *CI*) ranging from 1.01 to 1.27. The *OR* of bone tumor for an increase of 500 gram of birth weight was 1.01 (95% *CI* 1.00–1.02; *p* = 0.048 for linear trend). Interestingly, individuals with high birth weight had a greater risk for osteosarcoma (*OR* = 1.22, 95% *CI* 1.06–1.40, *p =* 0.006) than those with normal birth weight. In addition, in the subgroup analysis by geographical region, elevated risk was detected among Europeans (*OR* = 1.14, 95% *CI* 1.00–1.29, *p =* 0.049). The present meta-analysis supported a positive association between high birth weight and bone tumor risk.

## 1. Introduction

Bone tumors are the fourth leading type of cancer death among individuals under 20 years old, and the five-year survival is 61.8% in Europe and 79.0% in the USA [1,2,3]. According to a report from the American Cancer Society, it is estimated that there will be 2970 new cases and 1490 deaths of cancer of the bones and joints in 2015. Since bone tumors occur mostly in children and adolescents, they will have a significant and long-term impact on the life quality of patients and their families [4,5]. Thus, bone tumors are posing a potential threat to public health. However, so far, the etiology of bone tumor still remains unclear, resulting in the slow progress in corresponding prevention and treatment measures for bone tumor.

Among the reported 20 more various subtypes of bone tumor, osteosarcoma (OS), Ewing sarcoma (EWS) and chondrosarcoma (CS) are the major diagnosed types [6]. It is reported the bone tumor is diagnosed most commonly during puberty, when both the growth and development of bone are at a rapid speed. Thus, it can be supposed that factors associated with growth and development might be related with the occurrence and development of bone tumor, and this hypothesis has been further confirmed by epidemiological studies [7,8]. Birth weight, as an important factor to reflect intrauterine growth and development, had been reported to be related with subsequent bone growth in puberty [7,8,9]. In addition, growth factors including Insulin-like growth factors (*IGFs*), an altered immune function and imprinting errors, which was found to increase the risk for the occurrence and development of cancer, had been reported to be excessive among individuals with high birth weight [10,11,12,13]. To date, high birth weight was detected in relation to several cancers [14,15,16,17,18] and a potential association between high birth weight and bone tumor risk was suggested. Previously, three studies evaluated the association between birth weight and bone tumor risk, including OS, EWS and combined bone tumor, but the conclusions suggested a non-significant relationship [19,20,21]. However, birth weight of cases with EWS had been reported to be lighter than controls by Hartley *et al.* [22]; this result however, was in contrast to that by Valery *et al.* [23]. In addition, another study indicated a significantly elevated risk for individuals with high birth weight to develop OS [24]. The conflicting conclusions might result from the rarity of bone tumor cases. When the sample size was not enough, the statistical power to gain the underlying answer would be low and the probability to make mistakes would be considerable.

Thus, to provide a theoretical basis for the prevention of bone tumor, a meta-analysis was performed to pool all relevant published data to investigate the relationship between high birth weight and bone tumor risk. To the best of our knowledge, this work was the first meta-analysis on high birth weight in relation to the risk for bone tumor.

## 2. Methods

### 2.1. Search Strategy

To identify relevant studies on the relationship between high birth weight and bone tumor risk, an electronic search was performed in Embase, PubMed and Web of Science prior to June 2015. Index terms, including *birth weight, birth size, bone, cancer, tumor, neoplasm, carcinoma* and *sarcoma*, were applied in the literature search in various combinations. Besides, additional checks were performed on reference lists from eligible studies for further relevant articles. The language of the included articles was restricted to English.

### 2.2. Inclusion and Exclusion Criteria

Studies were taken into account when they satisfied the following inclusion criteria: (1) the study design was observational; (2) the study explored the association between high birth weight and the risk for bone tumor; and (3) the study reported relative risks with 95% *CIs* or provided sufficient information to calculate. Studies of improper type, including editorials, letters, reviews and non-human research, were excluded. Our study was conducted according to the guidelines for meta-analysis (PRISMA) [25].

### 2.3. Data Extraction

Data was collected separately by two authors (Songfeng Chen and Lin Yang) on the basis of the guidelines for meta-analysis [25]. When discrepancies appeared, discussions with a third reviewer (Zengwu Shao) were used for adjudication. The following data was collected from included studies: name of the first author, geographical region, publication year, research period, type of cancer, number of cases and controls, as well as reported *ORs* with their 95% *CIs* for each category of birth weight. In addition, when adjusted risk estimates were provided in the articles, adjusted variables would also be collected for further evaluation. If different results were reported in the same study based on different statistical models, we would extract the data adjusted with more confounders.

### 2.4. Quality Assessment

Quality assessment was performed by two reviewers (Songfeng Chen and Lin Yang) based on the Newcastle-Ottawa Scale [26] for observational studies in meta-analysis. This scale allocated a total score of 9 points, considering the selection process, the comparability of included studies, the identification of exposure and the definition of outcomes. The estimated scores of the included studies would be used in a quality-effect model [27,28], a method of weighting studies with quality scores to reduce the biases resulting from low quality studies. The results from the quality-effect model were introduced to explore the sensitivity and stability of our conclusions.

### 2.5. Statistical Analysis

In this paper, the overall relationship between high birth weight and bone tumor risk were evaluated on the basis of comparisons between the highest category of birth weight and the reference group in the identified articles. Except for the researches by Gelberg *et al.* [20] (reference group: 1984–2977 g), Buckley *et al.* [21] (reference group: 0–2700 g) and Troisi *et al.* [24] (reference group: 0–3000 g), the reference groups of other studies were in the range of normal birth weight (2500–4000 g). When the study explored two separate datasets or conducted the investigation in various graphical regions, it would be divided into independent studies stratified by country. In addition, if more than one kind of bone tumor was reported, the study would be considered as independent reports according to the type of cancer. *ORs* and 95% *CIs* were introduced as the common measurement for the relationship between high birth weight and bone tumor risk.

Dose-response analysis was conducted to further explore the relationship between high birth weight and bone tumor risk. According to a previously described method [29], studies reporting detailed numbers of cases and control in each categories of birth weight would be included in the dose-response analysis. Gram was used as the common measurement of birth weight and other measurements of birth weight were transformed into gram. The mean or median birth weight in each group would be introduced as the corresponding dose of birth weight in the dose-response analysis [29].

In order to choose the best-fitted effect model, heterogeneity was first evaluated in our meta-analysis. If heterogeneity was acceptable and negligible, a fixed-effect modeling method would be used in the analysis, otherwise a random-effect modeling method would be suggested [28,29,30]. *I*^2^ statistic and Cochran *Q* test were applied to estimate the heterogeneity among the identified studies [31]. Within the Cochran *Q* test, heterogeneity was suggested when the *p* for significance was lower than 0.10. *I*^2^ values of 25.0%, 50.0%, and 75.0% were assigned for low, moderate, and high heterogeneity, respectively [28,29,31].

Subgroup analysis by geographical region, age, adjustments and cancer type were performed in this meta-analysis. Besides, in order to explore potential origins of heterogeneity, sensitivity analyses were also employed. As for the evaluation of publication bias, it was estimated by funnel plots and the tests of Begg [32] and Egger [33]. No publication bias was confirmed when the *p* value for significance was higher than 0.05. In our meta-analysis, quality-effect model was performed with MetaXL (version 2.2) [28,29]. Other analyses were performed with STATA (version 12.0). In our analysis, all *p* values were calculated to be two-sided, with the statistically significant level to be 0.05.

## 3. Results

### 3.1. Characteristics of the Included Studies

**Figure 1:**
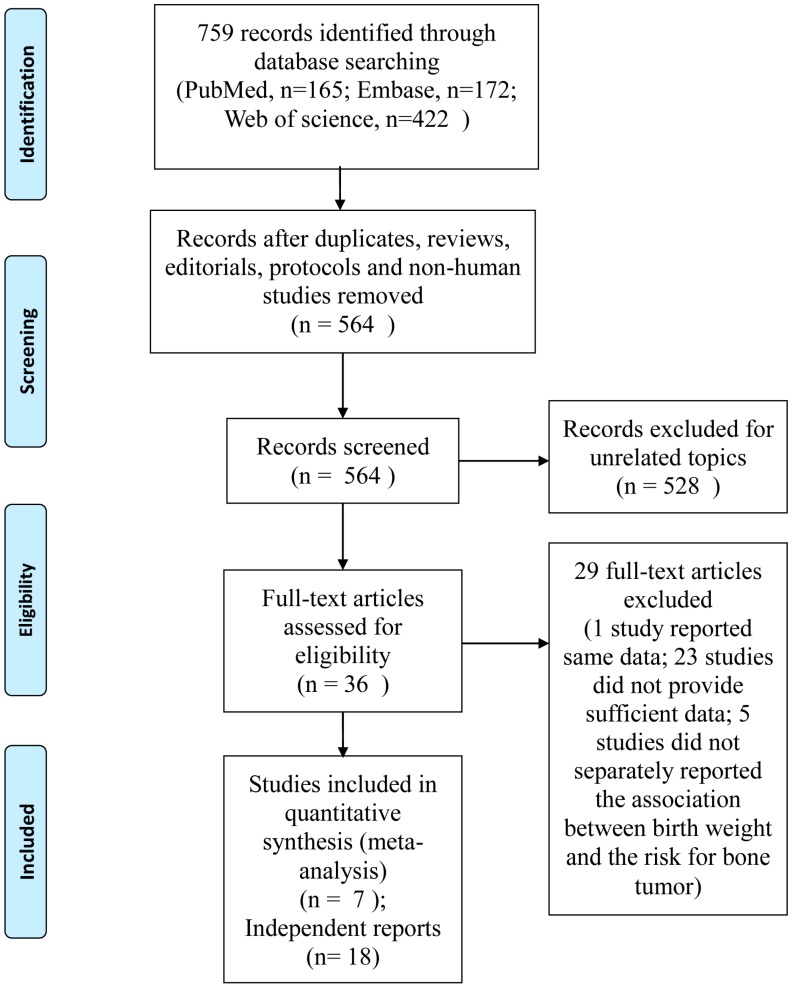
Flow chart of the identification and screening procedure.

The flow chart of the selection and identification process of our analysis was shown in Figure 1. After searching on Embase, PubMed and Web of Science, 759 studies were identified for further evaluation. Finally, seven case-control studies [14,20,21,24,34,35,36] were included according to our criteria. Among the seven studies, three [21,35,36] reported more than one kind of bone tumor, thus these studies were regarded as separate reports according to the type of cancer. In addition, one study [35] applied two independent datasets from two different countries and reported results separately. Therefore, this study [35] was divided into two separate studies. Under this circumstance, there were 18 independent reports to explore the potential relationship between high birth weight and bone tumor risk in our analysis.

General characteristics had been shown in the Supplementary File Table S1. In our study, the detailed numbers of cases and controls were not available in Bjorge *et al.* [34] and the controls were the same for cases with various kinds of bone tumor in O’Neill *et al.* [35] Thus, there were more than 2796 cases and 67,023 controls included in our analysis, containing more than 1864 cases and 12,748 controls from Europe and 932 cases and 54,275 controls from America. Among the 18 included studies, nine were performed on American population, while the other nine studies were conducted on a European population. Further, considering the major confounding effect of age, we extracted the data of age at diagnosis from the contained studies. Cutoff point of age was set to be 18 years old so as to explore the relationship between high birth weight and bone tumor risk in teenagers and mixed age groups, respectively. Twelve studies were conducted among teenage populations (age <18 years) and the age of research objects in the remaining six studies were below 45 years of age (mixed age group). In addition, types of cancer were also applied for further study. Among the included studies, two studies reported the results of combined bone tumor, while 13 studies evaluated the association in specific types of bone tumor including OS, EWS and CS. There were three studies that published the results of other specified and unspecified malignant bone tumors in addition to OS, EWS and CS; in our analysis, these results were classified into the group of other types of bone tumor. Quality assessment was conducted applying the Newcastle-Ottawa Scale. As shown in the Supplementary File Table S2, all the quality scores ranged from 4 to 8.

### 3.2. Association between High Birth Weight and Bone Tumor Risk

**Figure 2:**
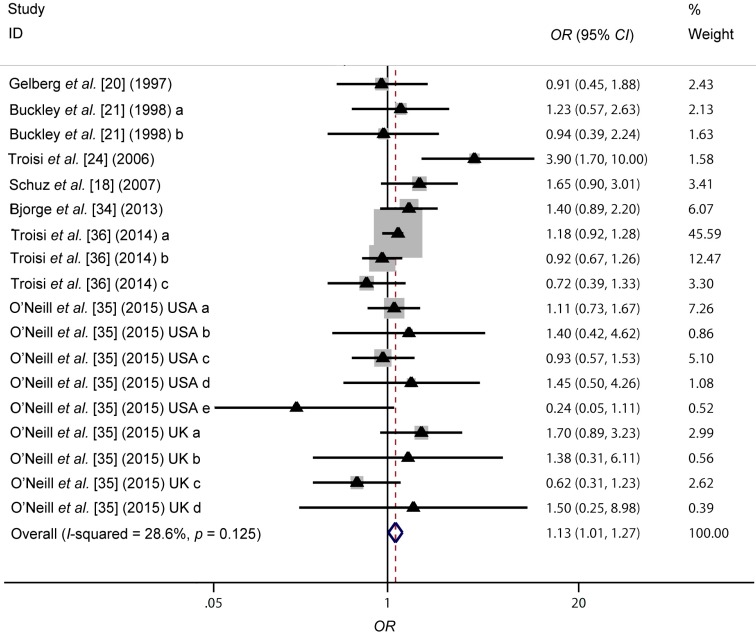
Forest plots of the fixed-effect model for the association of high birth weight and bone tumor risk. The horizontal lines and triangles denote the corresponding *ORs* and 95% *CIs*, while the vertical dotted line suggests the corresponding pooled *ORs*. The gray boxes point to the weight for each study. The open diamond corresponds to the overall *OR* and 95% *CI*.

According to the results, *I*^2^ value of 28.6% had demonstrated a moderate between-study heterogeneity. However, a *p* value for heterogeneity (*p*_h_) of 0.125 in Cochran’s *Q* test indicated that the heterogeneity was acceptable. Thus, a fixed-effect model was applied to evaluate the relationship between high birth weight and bone tumor risk and the forest plots of the results were displayed in Figure 2. A positive and significant relationship between high birth weight and bone tumor risk was found with an OR of 1.13 (95% CI 1.01–1.27, *p =* 0.029).

### 3.3. Dose-Response Analysis

**Figure 3:**
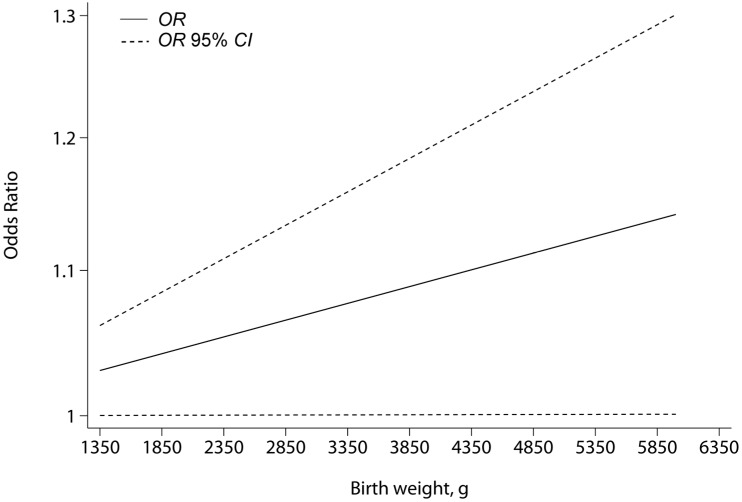
Results of dose-response analysis. The dotted lines denote the point-wise 95% *CIs*, while the solid line corresponds to *ORs* for the linear trend.

As for the dose-response analysis, a total of eight independent reports, including Schuz *et al.* [18] (2007), Gelberg *et al.* [20] (1997), Buckley *et al.* [21] (1998) a, Buckley *et al.* [21] (1998) b, Troisi *et al.* [24] (2006), Troisi *et al.* [36] (2014) a, Troisi *et al.* [36] (2014) b and Troisi *et al.* [36] (2014) c, providing adequate data satisfied the criteria. The results were shown in Figure 3. The *p* value of 0.935 for nonlinearity suggested the linear relationship between birth weight and the risk for bone tumor within the included studies. Thus, a linear model was suggested in this dose-response analysis. The summary OR of bone tumor for an increase of 500 gram of birth weight compared with the reference group was 1.01 (95% *CI* 1.00 to 1.02; *p =* 0.048 for linear trend).

### 3.4. Sensitivity Analyses

**Figure 4:**
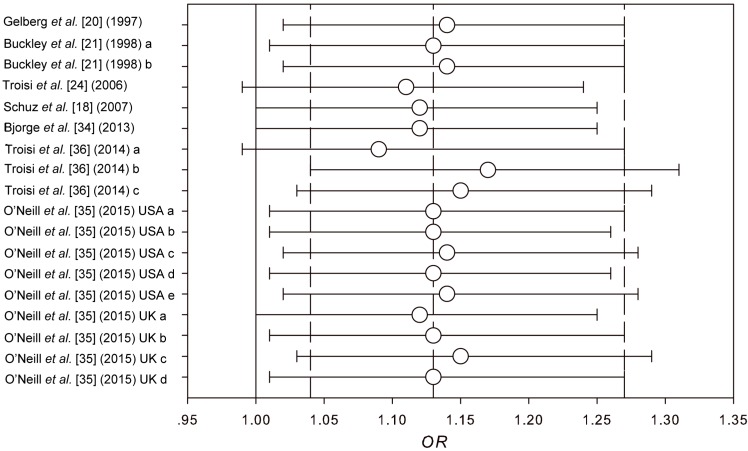
Results of leave-one-out method in sensitivity analysis. The three vertical dotted lines denote the pooled fixed effect of *OR* and 95% *CI* (*OR* = 1.13, 95% *CI* 1.01–1.27), while the solid vertical line shows the *OR* of 1. The horizontal lines and the circles indicate the *ORs* and 95% *CIs* applying the leave-one-out method.

Sensitivity analyses were conducted in our meta-analysis. Firstly, various modeling methods were introduced (fixed-effect model, *OR* = 1.13, 95% *CI* 1.01–1.27; random-effect model, *OR* = 1.13, 95% *CI* 0.96–1.33; quality-effect model, *OR* = 1.13, 95% *CI* 0.92–1.38). Though the results from these three models were similar, the association did not reach a significant level under the random-effect model and quality-effect model. Secondly, the leave-one-out method was conducted through eliminating one study successively. As shown in Figure 4, the conclusions was not drastically changed in this analysis and the ORs were in the range from 1.09 (95% *CI* 0.99–1.27) to 1.15 (95% *CI* 1.03–1.29). All the results were of significance or marginal significance.

### 3.5. Subgroup Analyses

**Figure 5:**
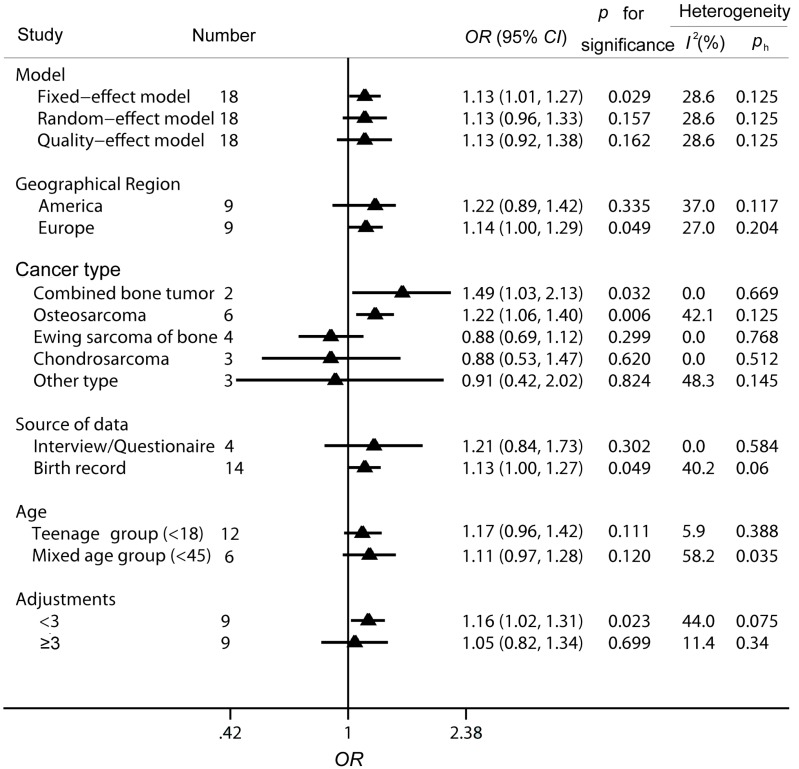
Subgroup analysis of *ORs* for bone tumor related to high birth weight. The term “≥3” means the study was adjusted by either age or sex.

To explore the potential between-study heterogeneity and evaluate the effects of various aspects on the association between high birth weight and the risk for developing bone tumor, subgroup analyses of geographical region, age, adjustments and cancer type were conducted. *ORs* and 95% *CIs* were extracted to pool the estimated risk and the outcomes were displayed in Figure 5. When divided by geographical region, there was a slightly increased association between high birth weight and bone tumor among the European population (*OR* = 1.14, 95% *CI* 1.00–1.29, *p =* 0.049), while a non-significant relationship was determined among the American population (*OR* = 1.22, 95% *CI* 0.89–1.42, *p =* 0.335). Only a marginal relationship was found in the age groups, both in the teenage group (*OR* = 1.11, 95% *CI* 0.97–1.28, *p =* 0.120) and mixed age group (*OR* = 1.17, 95% *CI* 0.96–1.42, *p =* 0.111). Further, it should be noted that heterogeneity was low in the mixed age group (*p*_h_ = 0.388, *I*^2^ = 5.9%). Considering adjustments as an important factor in estimating risks, we also extracted the data of adjustments adjusted to gain *ORs* and 95% *CIs* in the identified papers. Included studies were classified into two group. It was found that there was an increased association among the group with less than three adjustments (*OR* = 1.16, 95% *CI* 1.02–1.31, *p =* 0.023), while a non-significant relationship was detected among the group with three or more adjustments (*OR* = 1.05, 95% *CI* 0.82–1.34, *p =* 0.699). Subgroup analysis on the type of bone tumor was also performed for further evaluation. The reported data of bone tumor was divided into five groups, including groups of combined bone tumor, OS, EWS, CS and other type of bone tumors. Increased risk was found between high birth weight and bone tumor risk among the groups of combined bone tumor (*OR* = 1.49, 95% *CI* 1.03–2.13, *p =* 0.032) and OS (*OR* = 1.22, 95% *CI* 1.06–1.40, *p =* 0.006), while no association among the other three groups reached significant levels (EWS, *OR* = 0.88, 95% *CI* 0.69–1.13, *p =* 0.299; CS, *OR* = 0.88, 95% *CI* 0.53–1.47, *p =* 0.620; other type, *OR* = 0.91, 95% *CI* 0.42–2.02, *p =* 0.824). Notably, no heterogeneity was found among the groups of bone tumor (*p*_h_ = 0.669, *I*^2^ = 0.0%), or Ewing sarcoma of bone (*p*_h_ = 0.125, *I*^2^ = 0.0%) and chondrosarcoma (*p*_h_ = 0.512, *I*^2^ = 0.0%), which might partly explain the between-study heterogeneity.

### 3.6. Analysis on the Relationship between High Birth Weight and the Risks for the Three Main Subtypes of Bone Tumor in Age-Related Groups

**Figure 6:**
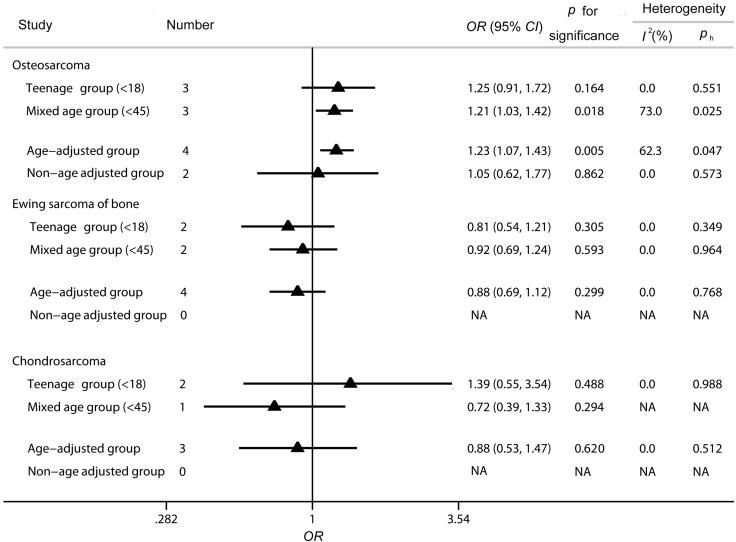
Analysis of the relationship between high birth weight and the risks for the three main subtypes of bone tumor in the age-related groups. “NA” means the data was non-available.

To further evaluate the relationship between high birth weight and the risks for the three main subtypes of bone tumor, OS, EWS and CS, we had conducted an analysis among age-related group. As shown from Figure 6, each subtype of bone tumor was divided into four groups, the teenage group, mixed age group, age-adjusted group and non-age adjusted group. As for the OS group, significant and positive association between high birth weight and OS risk was found among mixed age group (*OR* = 1.21, 95% *CI* 1.03–1.42, *p =* 0.018) and age-adjusted group (*OR* = 1.23, 95% *CI* 1.07–1.43, *p =* 0.005), while no evidence suggested the potential relationship within the teenage group (*OR* = 1.25, 95% *CI* 0.91–1.72, *p =* 0.164) and non-age adjusted group (*OR* = 1.05, 95% *CI* 0.62–1.77, *p =* 0.862). With regard to EWS and the CS group, all reported data was adjusted with age. However, no significant association was indicated between high birth weight and the risks for EWS (teenage group, *OR* = 0.81, 95% *CI* 0.54–1.21, *p =* 0.305; mixed age group, *OR* = 0.92, 95% *CI* 0.69–1.24, *p =* 0.593; age-adjusted group, *OR* = 0.88, 95% *CI* 0.69–1.12, *p =* 0.299) and CS (teenage group, *OR* = 1.39, 95% *CI* 0.55–3.54, *p =* 0.488; mixed age group, *OR* = 0.72, 95% *CI* 0.39–1.33, *p =* 0.294; age-adjusted group, *OR* = 0.88, 95% *CI* 0.53–1.47, *p =* 0.620) among the all four subgroups.

### 3.7. Publication Bias

**Figure 7:**
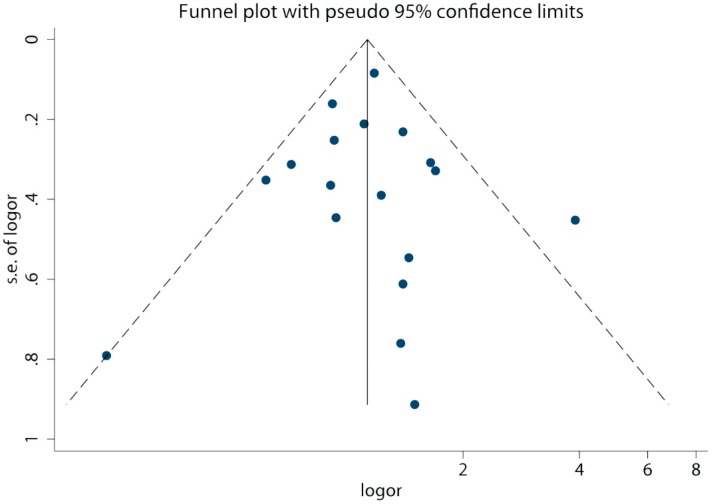
Funnel plot for studies on the relationship between high birth weight and bone tumor risk. The dashed lines are pseudo 95% confidence limits. The x-axis represents the *OR* on a logarithmic scale, while the y-axis points to the standard error of the *OR* on a logarithmic scale.

The results of the funnel plot analysis for publication bias, where little asymmetry was found, had been shown in Figure 7. Further, at the significance level of 0.05, no evidence for publication bias was observed in either the Begg or Egger tests (Begg, *p =* 0.910; Egger, *p =* 0.964); therefore, no publication bias was confirmed in our analysis.

## 4. Discussion

Since bone tumor incidence tends to increase around puberty, many investigators had suspected that bone tumors might be related with growth and development factors [6]. As reported, *IGF1* levels, sex and growth hormones would reach their maximum during the period of adolescent growth spurt and puberty, which might contribute to the development of bone tumors. As an important birth factor to reflect the *in utero* growth and development, birth weight is related with subsequent bone development and growth trends in puberty. Several mechanism studies suggested that high birth weight might play an important part in the occurrence and growth of bone tumors [24,34,37,38,39,40]. It was found that the rapidly growing tissue was highly susceptible to carcinogenesis and involved in the pathology of bone tumor [41]. This susceptibility to carcinogenesis could be due to the elevated vulnerability to carcinogenic substance, increase of mitotic errors or neoplastic transformation, resulting from the rapidly proliferating osteogenic cells [42,43]. Additionally, the positive relationships between high birth weight and the excess of *IGFs* was suggested to play an important role in cancer development [10,44,45]. It is plausible that individuals with high birth weight would be exposed to extra growth factors, leading to higher probability for the occurrence and development of cancer [46]. In recent years, a great number of studies on the relationship between high birth weight and the risk for bone tumor have been conducted, but the conclusions were inconsistent. Because of the rarity of bone tumors, the sample size among separate epidemiologic study on bone tumors was small, thus the statistical power was inadequate. In this paper, we included sixteen studies, containing more than 2796 cases with bone tumors. To date, to the best our knowledge, the present analysis was the first and the most comprehensive study to evaluate the relationship between high birth weight and the risk for bone tumor.

Meta-analysis has the advantage of clarifying the association with a larger power than that from separate studies. However, potential bias should be taken seriously in the meta-analysis [28]. Within the group by source of data, the birth record group (*OR* = 1.13, 95% *CI*, 1.00 to 1.27) possessed a lower risk than the interview/questionnaire group (*OR* = 1.21, 95% *CI*, 0.84 to 1.73), which might be due to the different recall bias. As for the interview/questionnaire group, interviewees might overstate the actual birth weight, when considering the high birth weight as an increased risk for bone tumor. Besides, recall bias would occur resulting from long time lag between birth time and disease diagnosis. In contrast, the data from birth records or birth registries should have no recall bias; thus, conclusions from the birth record group were assumed to be more reliable. In addition to the analyses on the source of data, selection and investigation bias were also considered using the Newcastle-Ottawa Scale. The estimated score ranged from four to eight, indicating the good quality of the included studies. In addition, the results of publication bias suggested that there was no evidence of publication bias in our analysis, thus the method of pooling published data was reasonable.

Though the etiologies of bone tumors are similar, they are different in many aspects [47,48]. Thus, combining various types of bone tumor would lead to problems, including potential heterogeneity between studies. In our meta-analysis, *I*^2^ value of 28.6% and *p*_h_ of 0.125 had indicated that the heterogeneity was moderate but acceptable. To evaluate the potential source of heterogeneity, subgroup analysis on type of cancer was performed. In our meta-analysis, there were two studies on combined bone tumor, six studies on OS, four studies on EWS, three studies on CS and three studies on other types of bone tumor. As seen in Figure 5, significantly increased association was only found among the bone tumor group (*OR* = 1.49, 95% *CI* 1.03–2.13) and OS group (*OR* = 1.22, 95% *CI* 1.06–1.40), while no increased risk for bone tumor was detected among the EWS group, CS group and other type group. The significant and positive relationship between high birth weight and OS risk might be ascribed to the following reasons. On one side, birth weight could reflect intrauterine growth and development and might predict a pubertal growth spurt or rapid bone growth during adolescence [21]. Birth weight was a good indicator to evaluate pre and postnatal development, which also had important short and long-term implications on health and survival. Clinical observations and epidemiological incidence patterns of osteosarcoma in humans and animals particularly, suggest a link with growth to onset of osteosarcoma [21,49]. On the other side, the association between birth weight and *IGF*-I was suggested [50,51]. High birth weight infants tended to display high circulating levels of *IGF*-I. *IGF*-I was involved in normal bone growth and differentiation, and the association between the growth of OS cell and the modulation of *IGF*-I is reported in many molecular biology experiments [52,53]. The biological effect of *IGF*-I and other growth factors might be ascribed to the findings in our study. Previously, a pooled analysis on the association between birth weight and OS risk with 434 cases was conducted by Mirabello *et al.* [54]. It was reported that individuals with high birth weight possessed an increased risk for OS (*OR* 1.35, 95% *CI* 1.01–1.79). In our analysis on the OS group, there were 1571 cases included and the results in our analysis was in agreement with the previous research, further demonstrating the positive association between high birth weight and OS risk. However, the potential relationship among the EWS group and CS group should not be denied because the sample size was much smaller than that of OS group. For CS group, since incidence of CS rose with age, and occurred rarely in childhood, there were only 123 cases included to evaluate the association among the CS group. With regard to the EWS group, 937 cases were eligible in our analysis.

Bone tumors are reported to be common among childhood and adolescent individuals, and they were more likely to occur among males than females [2]. Thus, age and sex were major confounders on the association between high birth weight and bone tumor risk. However, in our study, the results from subgroup analysis by age had suggested that there was only marginal effect found in both of the teenage group and mixed age group. It could be implied that the association between high birth weight and the risk for bone tumor was not age-distributed, unlike the age distribution of the incidence of bone tumor, but occurred in the whole age group. Since data of the sex distribution was not available in most of the included studies, we could just evaluate the confounding effect of sex through the subgroup analysis on the number of adjustments. Consequently, the group with more adjustments (*OR* = 1.05, 95% *CI* 0.82–1.34) had a lower risk than the group with less adjustments (*OR* = 1.16, 95% *CI* 1.02–1.31), but failed to reach a significant level. Generally, the results adjusted with more confounders were thought to be more reliable. According to our results, the potential association between high birth weight and the risk for bone tumor among the overall population was not stable and robust. However, we should note that in this subgroup analysis by adjustments, the confounding effect of age, sex and other potential confounders have been mixed together, leading to unexpected outcomes.

As for the major confounding factor of age, attention is deserved since various subtypes of bone tumor were related to the different age distribution. It had been reported that Ewing’s sarcoma and OS were the most frequent histologic subtypes in the first two decades, while CS showed an increased incidence after the fourth decade [48]. Particularly, CS incidence rose with age and occurred rarely in teenagers. Thus, we should further evaluate the relationship between high birth weight and the risks for the three main subtypes of bone tumor in the age-related groups. As seen from Figure 6, each subtype of bone tumor was divided into four groups, respectively. For the OS group, no significant relationship was found among the teenage group (*OR* = 1.25, 95% *CI* 0.91–1.72, *p =* 0.164), while a positive and significant association was detected among the mixed age group (*OR* = 1.21, 95% *CI* 1.03–1.42, *p =* 0.018). Though the incidence of OS was reported to be more common among younger individuals, the results suggest that the relationship between high birth weight and bone tumor risk was not age-distributed. In addition, the influence of age was further investigated depending on whether age was adjusted or not in the original studies. Consequently, there was a significant and positive association between high birth weight and bone tumor risk after the age at diagnosis was adjusted (*OR* = 1.23, 95% *CI* 1.07–1.43, *p* = 0.005), while no significant relationship was detected within the non-age adjusted group (*OR* = 1.05, 95% *CI* 0.62–1.77, *p* = 0.862). The combined results from age-adjusted data were more reliable and convincing. The results in this analysis further confirmed the positive association between high birth weight and the risk for OS. For ES and CS subtype group, all the included studies were adjusted with age, but the results suggest there was no significant relationship confirmed among all the groups, as shown in Figure 6.

Genetic factors were reported to play an essential part in the occurrence and development of bone tumor. As reported by Savage *et al*., tumor protein p53 (*TP53)* germ line mutations was found to be related with OS [6,55]. When it came to EWS, low incidence was found among African and Chinese populations [6,56,57,58], suggesting the potential interaction between genetic factors and bone tumor risk. According to epidemiology studies, familial diseases were found to be in relation to the risk of bone tumor [59,60,61]. Besides, polymorphisms in important growth factors including *IGF* had been indicated to be involved in the interaction between high birth weight and bone tumor risk. In our analysis, subgroup by geographical region was conducted, with a significantly increased risk found among the European group (*OR* = 1.14, 95% *CI* 1.00–1.29) but not the group from America (*OR* = 1.22, 95% *CI* 0.89–1.42). These results might be due to the different ethnic constitutions of these two groups. It was reported that incidence of bone tumors, especially for EWS and CS, are more common in Caucasians than Blacks [62]. However, it should be noticed that the sample size of the American population is not comparative with that of the European population. In our study, there were more than 2796 cases and 67,023 controls included in our analysis, containing 1864 cases and 12,748 controls from Europe and 932 cases and 54,275 controls from America. According to the reported data [47], the adjusted incidence rate for all bone and joint malignancies is 0.9 per 100,000 persons per year. Based on the National Vital Statistics Reports on birth data [63], the estimated frequencies indicate that high birth weight represents anywhere from 3.5% to 10.0% of all births. Thus, when the frequency of high birth weight is set to be 7.0%, our meta-analysis has a power of 40.2% to detect an *OR* of 1.13 for the association between high birth weight and the risk for bone tumor among the overall population. As for the European population, the power was calculated to be 26.8%, while the power among the America group was 18.2%. Therefore, the small statistical power due to the small sample size might contribute to the different findings between Europe and America groups. More relevant studies on American populations are required to increase the power for reliable conclusions.

Several limitations should be noted in the present meta-analysis. First, since relevant studies were limited, major confounding factors, including gestation period, dietary factors detailed data on sex and environmental exposure factors, were unable to be studied. Second, only published data was included in our electronic search, which could lead to the potential publication bias. Third, there were only American and European populations included in our meta-analysis, which might prevent our conclusions from extending to Asian and African populations. Relevant studies on Asian and African population are needed for further evaluation.

## 5. Conclusions

In the present analysis, 18 independent studies with more than 2796 cases were included. The results indicated positive and significant relationships between high birth weight and bone tumor risk. Further, individuals with high birth weight were found to be more likely to develop OS, while European populations with high birth weight exhibited a greater risk for bone tumors. While the sample size of our meta-analysis was sufficient, caution is required due to the inevitable heterogeneity. Additional studies of the biologic mechanisms underlying this relationship are necessary to allow more definitive and reliable conclusions.

## Supplementary Files

Supplementary File 1
