# Supplementary material for: High Birth Weight Increases the Risk for Bone Tumor: A Systematic Review and Meta-Analysis
Source: Int J Environ Res Public Health. 2015 Sep 9;12(9):11178–95. doi: 10.3390/ijerph120911178 (PMC4586668; doi:10.3390/ijerph120911178)
Supplement: Supplementary File 1 [file ijerph-12-11178-s001.pdf]

High Birth Weight Increases the Risk for Bone Tumor: A Systematic Review and Meta-Analysis

Table S1. Characteristics of the included studies on association between high birth weight and bone tumor risk.

Study	Year	Country	Period	Source of data	Age	Cancertype	Case/ control	Birth weight	*OR* (95% *CI*)	Adjustment	QA
Gelberg 1997	1997	USA	1978–1988	Interview/Questionaire	<25	Osteosarcoma	126/126	1984–2977g<br>2978–3313g<br>3314–3664g<br>3665+g	1.00(reference)<br>1.95（0.93–4.07）<br>1.20（0.61–2.37）<br>0.91（0.45–1.88）	Crude	7
Buckley 1998 a	1998	US and Canada	1982–1987	Interview/Questionaire	<18	Osteosarcoma	151/150	0–2700g<br>2800–3200g<br>3200–3600g<br>3600+g	1.00(reference)<br>0.84（0.38–1.88）<br>0.81（0.38–1.72）<br>1.23（0.57–2.63）	Crude	4
Buckley 1998 b	1998	US and Canada	1982–1987	Interview/Questionaire	<21	Ewing sarcoma of bone	152/153	0–2700g<br>2800–3200g<br>3200–3600g<br>3600+g	1.00(reference)<br>1.46（0.59–3.65）<br>0.85（0.36–1.98）<br>0.94（0.39–2.24）	Crude	4
Troisi 2006	2006	USA	1994–2000	Birth records	<39	Osteosarcoma	144/130	3000–3499g<br>3500–3999g<br>4000+g	1.00(reference)<br>1.30（0.73–2.40）<br>3.90（1.70–10.00）	Sex and age	6
Schuz 2007	2007	German	1992–1997	Interview/Questionaire	<17	Combined bone tumor	95/2577	2500–4000g<br>4000+g	1.00(reference)<br>1.65（0.90–3.01）	Gender and age at diagnosis	7
Bjorge 2013	2013	Denmark Finland, Norway, Sweden	1967–2010	Birth registry	<15	Combined bone tumor	NA	3000–3499g<br>3500–3999g<br>4000–4499g<br>4500–6000g	1.00(reference)<br>1.10（0.85–1.30）<br>1.20（0.91–1.60）<br>1.40（0.89–2.20）	Gestational age, birth weight, and maternal age	8

Table S1. *Cont.*

Study	Year	Country	Period	Source of data	Age	Cancertype	Case/ control	Birth weight	*OR* (95% *CI*)	Adjustment	QA
Troisi 2014 a	2014	Norway, Sweden, Denmark	1970–2010	Birth recordss	<45	Osteosarcoma	509/5088	2500–3999g<br>4000+g	1.00(reference)<br>1.18（0.92–1.28）	Birth year and sex	8
Troisi 2014 b	2014	Norway, Sweden, Denmark	1970–2010	Birth records	<45	Ewing sarcoma of bone	305/3042	2500–3999g<br>4000+g	1.00(reference)<br>0.92（0.67–1.26）	Birth year and sex	8
Troisi 2014 c	2014	Norway, Sweden, Denmark	1970–2010	Birth records	<45	Chondrosarcom a	98/984	2500–3999g<br>4000+g	1.00(reference)<br>0.72（0.39–1.33）	Birth year and sex	8
O'Neill 2015 USA a	2015	USA	1985–2004	Birth records	28d–14y	Osteosarcoma	251/53716	3000–3499g<br>3500–3999g<br>4000+g	1.00(reference)<br>0.95（0.70–1.30）<br>1.11（0.73–1.67）	Sex, period' region of birth，gestational age, birth order, plurality, maternal age and race/ethnicity	7
O'Neill 2015 USA b	2015	USA	1985–2004	Birth records	28d–14y	Chondrosarcom a	13/53716	3000–3499g<br>3500–3999g	1.00(reference)<br>1.40（0.42–4.62）	Sex, period' region of birth, gestational age, birth order, plurality, maternal age and race/ethnicity	7
O'Neill 2015 USA c	2015	USA	1985–2004	Birth records	28d–14y	Ewing sarcoma of bone	202/53716	3000–3499g<br>3500–3999g<br>4000+g	1.00(reference)<br>1.22（0.87–1.81）<br>0.93（0.57–1.53）	Sex, period' region of birth，gestational age, birth order, plurality, maternal age and race/ethnicity	7

Table S1. *Cont.*

Study	Year	Country	Period	Source of data	Age	Cancertype	Case/ control	Birth weight	OR (95% CI)	Adjustment	QA
O'Neill 2015 USA d	2015	USA	1985–2004	Birth records	28d–14y	Other specified malignant bone tumours	31/53716	3000–3499g<br>3500–3999g<br>4000+g	1.00(reference)<br>1.13（0.48–2.69）<br>1.45（0.50–4.26）	Sex, period' region of birth, gestational age, birth order, plurality, maternal age and race/ethnicity	7
O'Neill 2015 USA e	2015	USA	1985–2004	Birth records	28d–14y	Unspecified malignant bone tumours	14/53716	3000–3499g<br>3500–3999g	1.00(reference)<br>0.24（0.05–1.11）	Sex, period' region of birth, gestational age, birth order, plurality, maternal age and race/ethnicity	7
O'Neill 2015 UK a	2015	UK	1980–2007	Birth records	0–14y	Osteosarcoma	390/583	3000–3499g<br>3500–3999g<br>4000+g	1.00(reference)<br>1.01（0.70–1.46）<br>1.70（0.89–3.23）	Sex, period and region of birth	8
O'Neill 2015 UK b	2015	UK	1980–2007	Birth records	0–14y	Chondrosarcom a	12/17	3000–3499g<br>3500–3999g	1.00(reference)<br>1.38（0.31–6.11）	Sex, period and region of birth	8
O'Neill 2015 UK c	2015	UK	1980–2007	Birth records	0–14y	Ewing sarcoma of bone	288/436	3000–3499g<br>3500–3999g<br>4000+g	1.00(reference)<br>0.84（0.54–1.30）<br>0.62（0.31–1.23）	Sex, period and region of birth	8
O'Neill 2015 UK d	2015	UK	1980–2007	Birth records	0–14y	Other specified malignant bone tumours	15/21	3000–3499g<br>3500–3999g	1.00(reference)<br>1.50（0.25–8.98）	Sex, period and region of birth	8

OR odds ratio, *CI* confidence internal, *QA* quality assessment, *NA* not available.

Table S2. Quality assessments of the identified case–control studies on high birth weight in relation to bone tumor.

Study	Selection			Comparability			Exposure			Overall Quality Score
	1	2	3	4	5	6	7	8	9	
	Indicates cases independently validated	cases are representative of population	community controls	controls have no history of bone tumor	study controls for age	study controls for additional factor	ascertainment of exposure by blinded interview or record	same method of ascertainment used for cases and controls	nonresponse rate the same for cases and controls	
Gelberg 1997	1	1	1	0	1	1	1	1	0	7
Buckley 1998 a	1	1	0	0	1	0	0	1	0	4
Buckley 1998 b	1	1	0	0	1	0	0	1	0	4
Troisi 2006	1	0	0	0	1	1	1	1	1	6
Schuz 2007	1	1	1	0	1	1	0	1	1	7
Bjorge 2013	1	1	1	1	1	1	1	1	0	8
Troisi 2014 a	1	1	1	1	1	1	1	1	0	8
Troisi 2014 b	1	1	1	1	1	1	1	1	0	8
Troisi 2014 c	1	1	1	1	1	1	1	1	0	8
O'Neill 2015 USA a	1	1	1	0	1	1	1	1	0	7
O'Neill 2015 USA b	1	1	1	0	1	1	1	1	0	7
O'Neill 2015 USA c	1	1	1	0	1	1	1	1	0	7
O'Neill 2015 USA d	1	1	1	0	1	1	1	1	0	7
O'Neill 2015 USA e	1	1	1	0	1	1	1	1	0	7

Table S2. *Cont.*

Study	Selection			Comparability			Exposure			Overall Quality Score
	1	2	3	4	5	6	7	8	9	
	Indicates cases independently validated	cases are representative of population	community controls	controls have no history of bone tumor	study controls for age	study controls for additional factor	ascertainment of exposure by blinded interview or record	same method of ascertainment used for cases and controls	nonresponse rate the same for cases and controls	
O'Neill 2015 UK a	1	1	1	1	1	1	1	1	0	8
O'Neill 2015 UK b	1	1	1	1	1	1	1	1	0	8
O'Neill 2015 UK c	1	1	1	1	1	1	1	1	0	8
O'Neill 2015 UK d	1	1	1	1	1	1	1	1	0	8
O'Neill 2015 UK e	1	1	1	1	1	1	1	1	0	8

The study quality was assessed according to the Newcastle Ottawa Quality assessment scale for case–control studies. This scale awards a maximum of 9 points to each study: 4 for selection, 2 for comparability, and 3 for assessment of outcomes (for cohort study). 1 = "Yes", 0 = "No", "Unable to determine" or "Not available". For case–control studies, 1, indicates cases independently validated; 2, cases are representative of population; 3, community controls; 4, controls have no history of bone tumor; 5A, study controls for age; 5B, study controls for additional factor(s); 6, ascertainment of exposure by blinded interview or record; 7, same method of ascertainment used for cases and controls; and 8, nonresponse rate the same for cases and controls.

© 2015 by the authors; licensee MDPI, Basel, Switzerland. This article is an open access article distributed under the terms and conditions of the Creative Commons Attribution license (http://creativecommons.org/licenses/by/4.0/).
